# Geographic variation and risk factors for teenage pregnancy in Uganda

**DOI:** 10.4314/ahs.v20i4.48

**Published:** 2020-12

**Authors:** Joseph Byonanebye, Ruta Brazauskas, Nazarius Tumwesigye, Staci Young, Thomas May, Laura Cassidy

**Affiliations:** 1 Marquette University, College of Health Sciences; 2 Medical College of Wisconsin, Institute of Health and Equity; 3 Makerere University, School of Public Health; 4 Medical College of Wisconsin, Department of Family and Community Medicine; 5 Washington State University, Elson S. Floyd College of Medicine; 6 Medical College of Wisconsin, Institute of Health and Equity

**Keywords:** Teenage pregnancy, risk factors, Uganda demographic, health survey

## Abstract

**Background:**

Teenage pregnancy is a global health issue with high rates in sub-Saharan Africa. In Uganda, teenage pregnancy is a public and community health issue.

**Objectives:**

This study hypothesized that there would be regional variations in rates, risk factors and trends of teenage pregnancy in Uganda.

**Methods:**

Data were analyzed from the Uganda Demographic and Health Surveys (UDHS) in 2006 and 2011. The outcome of interest was current pregnancy for females 15 to 19 years of age at the time of the survey. Bivariate analysis was performed for each year to examine the rate and trends of pregnancy by various demographic characteristics. Logistic regression was conducted to assess the association between teenage pregnancy and sociodemographic variables.

**Results:**

Uganda's rate of teenage pregnancy increased from 7.3/1000 in 2006 to 8.1/1000 in 2011. The East Central region consistently had the highest rates than other regions. In 2006, teenage pregnancy was significantly associated with being married, living with a partner or separated, as compared to those who were single. Marital and wealth status were also significant predictors of teenage pregnancy based on the 2011 survey.

**Conclusion:**

The rate of teenage pregnancy in Uganda is high and the trend demonstrated regional variation. Future interventions could focus on regions with high poverty and low education.

## Introduction

Teenage pregnancy is a global health issue. According to the World Health Organization (WHO), over 16 million teenagers give birth every year, and more than 95% of those live in low and middle-income countries. The Millennium Development Goals (MDGs) were established in 2000 by 142 countries to guide and measure the progress towards a shared vision of reducing poverty and promoting wellbeing.^1^ Adolescent health, reducing maternal mortality and, achieving universal reproductive health are three of the MDGs.^2^ The four indicators that measure health in this context are . Currently, Sub-Saharan Africa lags behind in the adolescent sexual reproductive health disparities goals.^2^

In Uganda, teenage pregnancy is a public and community health issue of paramount importance because of the country's low social economic status and population structure. Fifty two percent of the population is below 18 years of age and approximately 25 percent of teenage girls become pregnant, a proportion that ranks Uganda higher than the other East African countries.^3^ Girls who are from low socioeconomic backgrounds, especially those living in rural areas, are highly affected by poverty, which could escalate the disparities that exist between social classes. The school dropout rate for girls is significantly attributed to teenage pregnancy, thus limiting their future financial capacity.^4^,^5^

The myriad health consequences of teenage pregnancy can affect both the mother and the baby.^6^,^7^ Teenage pregnancy is linked to higher rates of maternal and infant mortality. According to the World Health Organization (WHO), teenage pregnancy and subsequent child birth at an early age accounts for 23% of the global burden of ill health as identified through disability adjusted life years.

Pregnant Ugandan teenagers are stigmatized and less likely to be accepted within their families and communities.^8^. In addition, teenagers have unmet medical and counselling needs.^9^,^10^ The need is much higher among those that live in the rural areas.^9^ Access to reproductive health services is limited by social stigma.^6^,^8^,^11^

In Uganda, there are inequalities in poverty, income and education at the regional level. In 1992, the central region had the highest income for both rural and urban populations according to the Uganda Bureau of Statistics. The northern region was the poorest with 75% of the rural population defined as poor while 50% of the urban population was also defined as poor. The northern region also has the lowest income at the household level 12 while the central region has the most educated household heads.

The geographic regions are occupied by different ethnic groups which have unique cultures. The Bantu dominate the central region and southern region, the central Sudanic live in the North West, and the Nilotic dominate the northern regions. Among the tribes, the Baganda dominate the central region, the Banyankole, Bakiga and Batoro dominate the western region, Basoga, Iteso and Bagisu dominate the eastern region, and the Langi, Acholi, Lugbra, Alur and Karamajong dominate the northern region. Given such diversity among and within the regions, there could be cultural variation in the attitudes toward pregnancy prevention.

The goal of this study was to evaluate the association between health, societal, demographic factors and the rates of teenage pregnancy in the diverse geographic parts of Uganda and the recent trends of teenage pregnancy in the nine regions.

## Materials and methods

**Figure 1:**
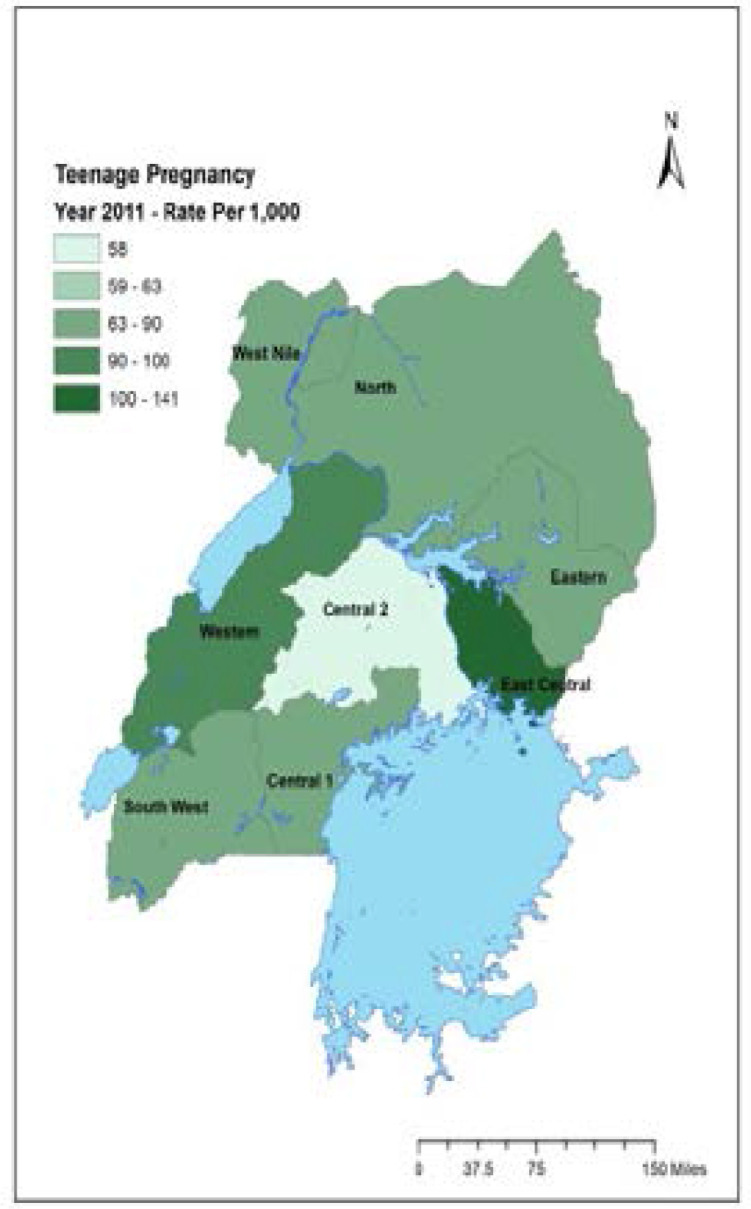
Map of Uganda showing teenage pregnancy rate/1000 for the year 2011 The East Central region had the highest rates of teenage pregnancy of 104/1000 while South West and Central II regions had the lowest rates.

**Fig 2:**
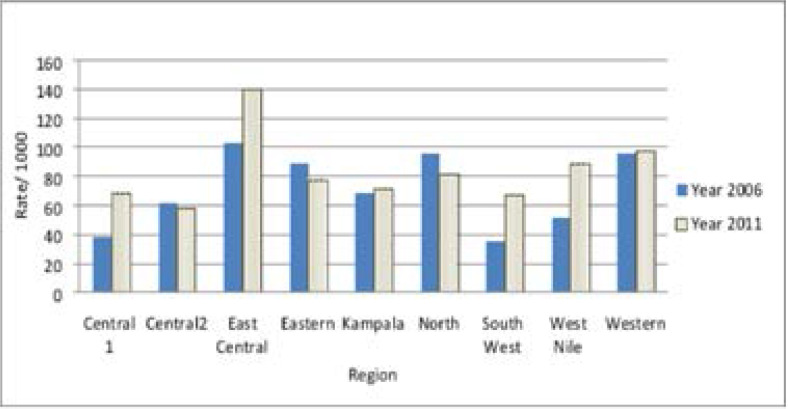
Comparing the rate of teenage pregnancy by region, Uganda Demographic and Health Survey, 2006 and 2011

The study was approved by the Medical College of Wisconsin Institutional Review Board and the Higher Degrees, Research and Ethics Committee of Makerere University, School of Public Health. A secondary analysis was conducted using the Uganda Demographic and Health Survey (UDHS) data sets for 2006 and 2011. The UDHS is a cross-sectional survey that samples each region of Uganda. The UDHS collects information on populations, health, HIV status, and nutrition from Uganda for the purpose of setting targets and making policies. The survey uses a two-stage cluster sampling, which generates a nationally representative sample of households. The UDHS sampling weights account for clustering and complex design. The regions in the UDHS 2006 and UDHS 2011 are defined as: West Nile, North, Karamoja, Eastern, Western, East Central, Central 1, Central 2 and Southwest (Figures 1 – 2). The analysis focused on data from a woman's questionnaire, which includes basic demographic and fertility characteristics of women aged 15 to 49. For the purposes of this study, data on the subset of women aged 15 to 19 years were analysed.

Statistical analyses were conducted to describe the women's characteristics of the two UDHS datasets for 2006 and 2011. The main outcome of interest was current pregnancy status for teenagers aged 15 to 19 at the time of the survey. Other data analyzed included age, births in the last five years, highest educational level achieved, type of residence, wealth index, region of residence, religion, desire for current pregnancy, marital status and recent sexual exposure. The proportion of teenagers who were pregnant was computed for each region. Bivariate analysis was performed for each year to examine the rate of pregnancy by various demographic characteristics. The factors significantly associated with teenage pregnancy in each region were determined by logistic regression. A backward elimination model selection procedure was conducted to identify statistically significant covariates to be included the final model. Statistical level of significance of 0.05 was used throughout. All tests are two-sided. Appropriate weighting was done to account for complex survey design. All statistical analyses were conducted using SAS 9.4 (SAS Institute, Cary, NC).

## Results

Figures 1 and 2 show the rate of teenage pregnancies in each region of Uganda by year. The overall rate of teenage pregnancy was 71.5 per 1000 in 2006 and 83.5 per 1000 in 2011. The highest rate of teenage pregnancy was in the East Central region, with 103 per 1000 in 2006 and 140.1 per 1000 in 2011 (Figure 2). Additionally, the East Central region showed the second highest change in rates of teenage pregnancy.

Conversely, the lowest rates of teenage pregnancy were found in the South West, with 36 per 1000 in the 2006 and 67 per 1000 in 2011. In addition, Central 1 and Central 2 regions consistently had lower rates of teenage pregnancy. Central 1 region had a teenage pregnancy rate of 39 per1000 in the 2006 and 68.8 per 1000 in 2011; Central 2 region had a rate of 61.5 per 1000 in 2006 and 58.5 in 2011. Central 2 region, however, had a smaller change in the rate of teenage pregnancy as compared to other regions which had consistently low rates.

Table 1 shows the overall and regional sociodemographic characteristics of teenagers who participated in the 2006 and 2011 surveys. Overall, there were 1,948 respondents in 2006 and 2,026 respondents in 2011. At the time of the survey, 7.3% of teenagers were pregnant in 2006 and 8.1% were pregnant in 2011. A total of 24.3% and 23.9% of teenagers were either pregnant or had given birth in the five years preceding the 2006 and 2011 surveys.

**Table 1:** Social demographic characteristics of the teenagers in the nine regions of Uganda, 2006 and 2011

Region	Central 1		Central 2		E. Central		Eastern		Kampala		North		S. West		W. Nile		Western		Total	
Year	06	11	06	11	06	11	06	11	06	11	06	11	06	11	06	11	06	11	06	11
Participants																				202
(N)	205	189	179	171	212	207	191	231	216	223	223	208	222	209	173	135	218	247	1948	6
Pregnant	3.9	5.8	6.1	6.9	10.4	14.0	9.9	7.8	6.9	7.2	9.6	8.2	3.6	6.7	5.2	8.9	9.6	9.7	7.3	8.1
Birth in last 5 yrs	10.7	20.5	24.6	17.5	19.8	24.2	24.1	22.9	16.2	16.1	23.8	17.3	13.1	17.7	17.9	12.6	17.4	22.3	17.4	18.5
Pregnant or given birth	14.1	22.8	29.1	21.7	25.9	31.4	31.4	28.1	21.3	22.0	32.2	24.0	14.4	22.5	21.4	19.3	25.7	29.6	24.3	23.9
Residence																				
Urban	100	31.6	15.1	27.5	8.9	17.9	6.3	13.9	13	100	3.6	24.0	8.6	16.7	8.7	5.9	7.8	36.8	7.8	70.3
Rural	0	68.4	85	72.5	91.5	82.1	93.7	86.1	87	0	96.4	76	91.4	83.3	91.3	94.1	92.2	63	92.2	29.7
Education																				
No	0.5	1.1	0	0.5	0.9	0	1	0.4	0.5	0.9	19	1.4	2.7	5.3	4	60.0	4.1	3.6	4.1	5.5
Primary	30.2	48.0	63.1	57.7	62.3	61.8	73.8	74.5	44.9	39.5	72.6	81.3	71.1	61.2	85.5	32.6	81.2	82.2	81.2	62.3
Secondary	69.3	50.9	36.9	41.8	36.8	38.2	25.1	25.1	54.6	59.6	8.4	17.3	26.1	33.5	10.4	7.4	14.6	14.2	14.6	32.2
Wealth Index																				
Poor	0	9.9	11.7	15.9	24.1	23.2	50.8	52.4	8.8	0	4.9	61.1	18.9	20.6	58.4	89.6	27.5	57.9	35.5	34.3
Middle	0.5	12.3	14.5	13.8	17.5	20.3	20.4	19.9	14.8	1.8	6	14.9	31.1	27.3	15	3.7	31.6	14.9	16.4	16.0
Rich	99.5	77.8	73.7	70.4	58.5	56.5	28.8	27.7	76.4	98.2	9	24	50	52.2	26.6	6.7	40.8	27.1	49.1	49.7
Marriage																				
Never	79.9	83.6	80.4	79.9	79.7	75.4	71.2	71.4	83.8	79.8	65.7	75.4	85.1	78.5	78.6	74.8	73.4	70.9	73.4	77.3
Married	6.9	4.7	6.1	6.9	11.3	6.3	14.7	15.1	6.9	2.7	18.9	10.6	7.7	6.2	15	10.4	17.4	10.1	17.4	8.1
Partner	11.6	9.9	12.8	11.6	8	15.5	12	11.3	6.5	14.3	9.6	10.6	4.5	9.6	2.9	14.8	5.5	13	5.5	11.5
Separated	1.6	1.8	0.6	1.6	0.9	2.9	2.1	2.2	2.8	3.1	5.7	3.4	2.7	5.7	3.5	0	3.7	6.1	3.7	3.1
Religion																				
Catholic	34.9	45.0	41.1	34.9	24.5	17.4	30.4	40.7	39.8	35.9	67.5	62.0	36	36.4	50.3	86.7	43.1	59.5	43.1	43.4
Protestant	25.4	14.0	28	25.4	40.1	31.4	36.6	35.1	21.3	21.1	21.4	24	55	36.4	32.9	7.4	38.1	13.8	38.1	26.9
Muslim	14.8	24.6	15.1	14.8	21.2	32.9	12.7	10.4	24.5	23.3	1.2	1	3.6	3.8	15	3	4.6	23.9	4.6	14.5
Pentecostal	20.1	14.0	11.2	20.1	12.7	16.4	8.4	13	12.5	17.9	8.1	12.0	2.3	13.4	1.2	3	6	2.4	6	12.7
Others	4.2	2.3	4.5	4.8	1.4	1.9	9.9	0.9	1.9	1.8	1.8	1.0	3.1	10.5	0.6	0	8.2	0.4	8.2	2.5

In 2006, the North region had the highest percentage of teenagers (19%) who never attained any education. Across all regions, most of the teenagers (81.2%) had attained primary education while 14.6% of teenagers attained secondary education. The lowest percentage of primary educational attainment (30.2%) occurred in Central 1 region, and the highest educational attainment occurred in West Nile (85.5%). The percentage of secondary educational attainment was lowest in the North (8.4%) and highest in Central I region (69.3%). The North, West Nile and Eastern regions had the highest percentage of teenagers in the poorest category respectively, while Central 1 and Kampala had the highest percentage of teenagers in the richest category. Generally, across all regions, most of the teenagers had never been married. The North and Eastern regions had the highest percentage of married teenagers. Across all the regions, Catholics (43.1%) were the majority, followed by Protestants (38.1%)Muslims (4.6%), and Pentecostals (6.0%), respectively.

In 2011, the West Nile region had the highest percentage of teenagers (60%) who had never attained primary education. However, in that same period, the majority of teenagers had attained primary and secondary education in all the regions. Attainment of primary education ranged from 33.6% in the West Nile region to 82.6% in the Western region, while attainment of secondary education ranged from 7.4% in the West Nile to 59.6% in Kampala. The West Nile also had the highest percentage of lower income teenagers while Kampala had the highest percentage of higher income teenagers. The percentage of married teenagers was lowest in the Central 2 region but highest in the Western region. The percentage of teenagers who were sexually active in the four weeks preceding the survey was highest in the Eastern region (21.2%) and the East Central region (20.2%) while the percentage was lowest in the Southwest region (16.7%) and the West Nile region (16.7%). Conversely, the percentage of teenagers who were abstaining was highest in the South West region (83.7%) and West Nile region (83.7%) and lowest in East Central region (79.8%) and Eastern region (78.8%). The majority were Catholics (43%), followed by Protestants (26.9%) whereas Muslims (14.5%) and Pentecostals (12.7%) were the minority. (Figure 1 and Figure 2)

Table 2 shows the results of the bivariate analysis of characteristics associated with teenage pregnancy for each study year. In 2006 factors significantly associated with teenage pregnancy included residence, education, marital status, wealth index and recent sexual exposure. Teenagers who lived in the rural areas had twice the odds of getting pregnant (Odds ratio, OR, 2.41, 95% confidence interval, CI, 1.32, 4.39) when compared to teenagers who lived in urban areas. Teenagers who had attended primary education were less likely to become pregnant (OR, 0.41, 95% CI, 0.22, 0.75) and teenagers who had secondary education were less likely to become pregnant (OR, 0.26, 95% CI, 0.13, 0.52) when compared to teenagers who had never attended school. The odds of getting pregnant among those who were married were almost 14 times higher compared to those who were never married (OR, 13.76, 95% CI, 8.69, 21.77).

**Table 2:** Bivariate analysis of factors associated with current teenage pregnancy, Uganda Demographic and Health Survey, 2006 and 2011

		2006			2011	
		Odds ratio of being pregnant			Odds ratio of being pregnant	
Characteristic	%	(95% CI)	P-Value	%	(95% CI)	P-Value
Residence						
Urban	3.7	Reference		5.4	Reference	
Rural	8.2	2.41 (1.32, 4.39)	0.004	9.2	1.73 (1.15, 2.61)	0.008
Education			<0.001			0.007
No	17.6	Reference		12.5	Reference	
Primary	7.7	0.41 (0.22, 0.75)	<0.001	8.9	0.67 (0.38, 1.17)	0.163
Secondary	5.2	0.26 (0.13, 0.52)	0.004	5.8	0.41 (0.22, 0.77)	0.006
Wealth Index			0.001			0.001
Rich	4.7	Referent		4.9	Referent	
Middle	7.2	1.59 (0.98, 2.59)	0.063	9.5	2.09 (1.32, 3.31)	0.002
Poor	11.1	2.54 (1.72, 3.76)	<0.001	12.1	2.64 (1.84, 3.80)	<0.001
Marriage			<0.001			0.001
Never	2.3	Reference		2.0	Reference	
Married	25.6	13.76 (8.69, 21.77)	<0.001	31.1	20.60 (12.73, 33.45)	<0.001
Living with partner	28.1	15.68 (9.52, 25.83)	<0.001	31.8	21.20 (13.40, 33.52)	<0.001
Separated	13.2	6.00 (2.55, 14.14)	<0.001	11.1	5.30 (2.30, 12.36)	<0.001
Religion			0.031			0.124
Catholic	8.7	Reference		8.6	Reference	
Protestant	6.5	0.72 (0.49, 1.07)	0.108	6.3	0.71 (0.46, 1.12)	0.142
Muslim	6.8	0.76 (0.45, 1.30)	0.316	9.9	1.15 (0.74, 1.79)	0.531
Pentecostal	4.8	0.50 (0.24, 1.07)	0.076	6.6	0.75 (0.44. 1.28)	0.296
Others	7.2	0.81 (0.33, 1.98)	0.644	15.7	2.00 (0.84, 4.74)	0.118
Region			0.035			0.088
Kampala	6.9	Reference		7.2	Reference	
Central 1	3.9	0.52 (0.21, 1.30)	0.160	5.8	0.81 (0.37, 1.79)	0.888
Central 2	6.1	0.86 (0.34, 2.23)	0.759	6.9	0.94 (0.40, 2.02)	0.600
East Central	10.4	1.54 (0.75, 3.15)	0.237	14.0	2.10 (1.10, 4.17)	0.033
Eastern	8.9	1.29 (0.59, 2.84)	0.523	7.8	1.10 (0.51, 2.40)	0.811
North	9.6	1.39 (0.70, 2.75)	0.347	8.2	1.13 (0.53, 2.41)	0.743
South West	3.6	0.50 (0.20, 1.27)	0.146	6.7	0.94 (0.41, 2.17)	0.881
West Nile	5.2	0.76 (0.35, 1.63)	0.473	8.9	1.27 (0.52, 3.13)	0.599
Western	9.6	1.44 (0.68, 3.08)	0.344	9.7	1.41 (0.69, 2.87)	0.347

In the 2011, the characteristics which were significantly associated with teenage pregnancy included marriage, residence, wealth, recent sexual exposure, and having given birth in the last five years. The odds of getting pregnant were 20 times higher among married teenagers (OR 20.6, 95% CI, 12.73, 33.45), 21 times higher among those who were living with partners (OR, 21.20, 95% CI, 13.40, 33. 52) and 5 times higher among those who were separated (OR, 5.30, 95% CI, 2.3, 12.36) when compared to the odds of getting pregnant among those who had never been married. Teenagers of poor households had twice the odds of getting pregnant (OR, 2.64, 95% CI, 1.84, 3.80), when compared to the teenagers from most affluent homes. Similarly, the odds of getting pregnant were twice as high among teenagers of middle-income group compared to those from the highest income category (OR, 2.09, 95% CI, 1.32, 3.31). Among teenagers who had not given birth in the previous five years, the odds of getting pregnant, were significantly lower than the odds of getting pregnant among those who had given birth in the previous five years (OR, 0.45, 95% CI, 0.34, 0.62). Among those who had attained secondary level education, the odds of getting pregnant were lower than the odds of getting pregnant among those who had never gone to school (OR, 0.39, 95% CI, 0.21, 0.75). Among those who lived in Eastern region, the odds of getting pregnant were twice the odds of those who lived in Kampala (OR, 2.10, 95% CI, 1.10, 4.17). (Table 2)

Table 3 illustrates the results of the multivariate logistic regression analysis of the factors associated with teenage pregnancy for 2006 and 2011. Based off the 2006 survey, the odds of getting pregnant were twice as high among teenagers who lived in rural areas compared to teenagers who lived in urban areas (OR: 2.20, 95% CI, 1.14, 4.14). The odds of teenage pregnancy were 40.5 times higher if they were married (OR 40.50, 95% CI, 23.42, 70.09), 51 times higher among teenagers living with partners (OR, 51.53, 95% CI,27.48, 96.68), and 24.70 times higher among teenagers who were separated (OR 24.70, 95% CI, 9.70, 63.06) as compared to those who have not been married.

**Table 3:** Multivariate analysis of factors associated with current pregnancy in Uganda for the year 2006 and 2011

	2006		2011	
Characteristic	Odds ratio of being pregnant		Odds ratio of being pregnant	
	(95 CI)	P- Value	(95% CI)	P -value
Residence		0.018		
Urban	Reference		Not Significant	
Rural	2.17 (1.14, 4.14)	0.018		
Wealth Index				0.008
Rich			Reference	
Middle	Not Significant		2.20 (1.25,3.92)	0.007
Poor			2.00 (1.17, 3.52)	0.012
Marriage		<0.001		<0.001
Never	Reference		Reference	
Married	40.50 (23.42, 70.09)	<0.001	50.90 (28.36, 91.36)	<0.001
Living with partner	51.53 (27.48, 96.68)	<0.001	48.30 (27.68, 84.42)	<0.001
Separated	24.73 (9.70, 63.06)	<0.001	15.40 (5.90, 40.08)	<0.001
Region				0.010
Kampala			Reference	
Central 1			0.86 (0.34, 2.17)	0.750
Central 2			0.77 (0.32, 1.85)	0.553
East Central	Not Significant		1.98 (0.90, 4.38)	0.090
Eastern			0.48 (0.19, 1.21)	0.121
North			0.53 (0.20, 1.41)	0.203
South West			0.63 (0.24, 1.65)	0.343
West Nile			0.43 (0.14, 1.31)	0.136
Western			0.65 (0.28, 1.54)	0.329
Births last 5 years				
Yes	Reference		Reference	
No	8.20 (4.75, 14.21)	<0.001	5.20 (3.24, 8.26)	<0.001

In 2011, the odds of getting pregnant were two times higher among teenagers in a middle income group (OR: 2.20, 95% CI, 1.25, 3.92) and two times higher among teenagers with a poor wealth status (OR 2.00, 95% CI, 1.17, 3.52) compared to teenagers of higher income group. The odds of getting pregnant were 50.90 times higher among teenagers who were married (OR 50.90, 95% CI, 28.36, 91.36), 48.30 times higher among teenagers who were living with partners (OR, 48.3, 95% CI, 27.68, 84.42), and 15.54 times higher among teenagers who were separated (OR 15.54, 95% CI, 5.90, 40.08). The odds of getting pregnant were 5.20 times higher among teenagers who had not given birth in the five years preceding the survey (OR, 5.20, 95% CI, 3.24, 8.26).

## Discussion

Analysis of Uganda's Demographic and Health Survey data indicates that the teenage pregnancy rates increased from 2006 to 2011; and there was regional variation. Marriage was the most significant factor associated with teenage pregnancy in both 2006 and 2011. This finding is consistent with other literature that suggests that early child marriage is one of the most significant explanatory variables for teenage pregnancy in Uganda.^13^, ^16^ In this study, based on the health problem analysis model, we raise question on the extent to which marriage is a determinant, a direct or indirect contributing factor for teenage pregnancy. What comes first, marriage, dropping out of school or teenage pregnancy? It is possible that girls are married off while still attending school and consequently become pregnant. Alternatively, girls could drop out of school because of various reasons such as lack of tuition funding for school which could force them to get married and consequently to become pregnant early. On the other hand, girls who intentionally or unintentionally become pregnant may be forced to marry to take care of their children.

Child Marriage is a barrier to girls' schooling and lack of education is both a risk factor and an outcome of child marriage.^17^ Bivariate analysis shows that not attending school and early child marriage are factors for teenage pregnancy. Marriage and dropping out of school, further enhancing teenage pregnancy could be connected. Keeping girls in school and providing them with sexual reproductive health education could reduce the likelihood that they will marry early or have children early. The type of school and access by girls could elevate risks for teenage pregnancy. For example, the men and boys that girls meet while walking long distances to and from school, or the ones they meet while at school, if there are sexual relationships, could risk the girls. Competing demands and the lack of consistent family and school support, discrimination of pregnant girls and teenage mothers at school and in their communities could reduce their school attendance and further increase their risk to more pregnancies.^17^

Often teenage pregnancy is blamed on girls alone. Yet, men and boys impregnant these girls. The role of men and boys is crucial in teenage pregnancy prevention. Various governmental and non-governmental organizations are advocating for an increased engagement of men and boys to end prevent teenage pregnancies. More evidence on the effectiveness of engaging of men and boys to prevnt teenage pregnancy is needed, however. A randomized controlled trial is assessing schoolbased relationship and sexuality education intervention focusing on young male perspectives. 18, 19

The Uganda Constitution prohibits marriage of girls aged below 18 years of age. However, child marriages can be a result of poverty since parents sometimes marry off their daughters to secure their financial future, and the limited access to education for girls restricts their options only to getting married.^14^ In addition, Uganda has laws and policies on rape, defilement and other forms of sexual violence; however, they are not enforced adequately. The Penal Code Act (2007) for example, criminalizes sex with girls below 18 years as a capital offense which is punishable by death sentence. There is a National strategy on Ending Child Marriage and Teenage Pregnancy, however implementation is inadequate.^20^ The strategy aims at: improving the policy and legal environment to protect teenagers; improving access to sexual reproductive health services; changing sociocultural norms which increase risky behaviours in the communities and empowering boys and girls with information for the purpose of ending child marriage and teenage pregnancy 18 Uganda is in the process of implementing a new education curriculum that includes sex education. 15 We need to rely on good quality evidence when developing public health policy and guidelines for educating young people with the intent to healthy behaviours that prevent teenage pregnancies. If sex education were to become more available in schools, its effectiveness should be assessed by using well designed analytical studies to ensure that policies are working as expected.

The findings that those living in the rural areas, having lower educational attainment and lower wealth status were at highest risk is consistent with other reports.^3^,^13^ Living in the rural areas was associated with higher chance of teenage pregnancy in 2006, while low wealth status was uniquely significantly associated with teenage pregnancy in 2011. As with Healthy People 2020^21^, identifying contextual factors that influence teen pregnancy and other adverse sexual health outcomes among vulnerable youth will be helpful to understand a pathway to teenage pregnancy that may otherwise be missed. Teen pregnancy is a complex and multilayered issue, each partner has a significant role to play in improving the health and well-being of teenagers in their communities establishing nontraditional partnerships to address determinants identified by community members and to enhance community-level teen pregnancy prevention activities. Further need to assess the link between social determinants of health and teenage pregnancy should be addressed. The World Health Organization (WHO) framework for Social Determinants of Health (SDHs) has indicated a vital way to reduce health disparities in pregnancy among young people.^22^ This includes actions at the policy level, at the health system level, at the family and community level and at the individual level to address determinants of teenage pregnancy. WHO's guidelines include preventing early marriage; preventing early pregnancy through sexuality education, increasing education opportunities and economic and social support programs; increasing the use of contraception and reducing coerced sex.^23^ Teenage pregnancy is frequently both behaviorally mediated and linked to multiple social factors such as individual factors, family, peers, school, neighborhoods, media, exposure as well as macro level factors such as economic forces, historical events, national politics, natural events, norms and cultural values.^24^, ^25^, ^26^ It is known that most tribes or ethnic groups, because of historical migration patterns, predominantly occupy different geographical regions and are differentiated by their cultures. Of interest, therefore, would be to assess the role played by different tribal cultures either as being protective or risky for teenage pregnancy. In the US, Healthy People 2020 utilizes information from Teen Pregnancy Prevention (TPP) Program, a national program that funds diverse organizations working to prevent teen pregnancy across the United States. The program provides grants to implement and evaluate new and innovative approaches for evidence-based teen pregnancy prevention programs. TPP builds the capacity of youth-serving organizations to implement, evaluate, and sustain evidence-based teen pregnancy prevention programs.^27^

The fact that there was a regional variation in teenage pregnancy rates may be associated with historical and current perspectives of the country. Like most other countries, is influenced by global systems such as migration, trade, travel, communication and social policies. The possible impact of oppression such as colonialism, sexism, global capitalism may have impact on the prevalence of poverty and patterns of educational attainment, socio cultural changes, which also influence teenage outcomes. These factors should be studied to assess as to whether the extent of such forces have affected some regions either positively or negatively more than the others. A fact from this study, for example, that the East Central region has had the highest rates of teenage pregnancy of 104/1000 while South West and Central II regions has had the lowest rates is surprising. (Figure 1). Central II region, where Luwero District is located, has had a history of civil wars in the 1980s. One would have expected that regions that previously were politically unstable to have higher rates of teenage pregnancy than the East Central region which has relatively not had civil wars. There could be protective factors that come with a region's history and consequently, governmental recovery efforts to overcome the past challenges.

## Limitations

The results are limited because the UDHS survey is not designed exclusively to collect data on teenage pregnancy and other risk sexual reproductive health factors which limits the amount of available information. Because of using UDHS data, the survey did not include pregnancy data on individuals less than 15 years of age, a particularly vulnerable group. The analysis for this study was done on the comparable UDHS 2006 and 2011 data alone. UDHS 2016 data, which could also be comparable, was available after this report was completed. Variables and geographical demarcations of the regions for the previous years in UDHS 1988–1989, 1995, 2000–2001 has not been consistent.

This study recommends program and policy interventions and further research. We recommend a national teenage pregnancy prevention program for Uganda. The program, for example, could be similar to the Teen Pregnancy Prevention (TPP) Program in the US, which funds diverse organizations working to prevent teen pregnancy. The program provides grants to implement and evaluate new and innovative approaches for evidence-based teen pregnancy prevention programs. (27) The program could guide the implementation of the National Strategy on Ending Child Marriage and Teenage Pregnancy to further protect the rights of girls against all forms of abuse and harmful traditional practices.^16^ To reduce regional variation of teenage pregnancy, prevention programs should focus on regions that are consistently having higher rates. We suggest in-depth to studies to assess the directional nature of early marriage and other determinants of teenage pregnancy. This study predicts that early marriage increases the risk for pregnancy. What comes first, marriage, dropping out of school or teenage pregnancy? This question should be answered to effectively prevent teenage pregnancies. Further exploration of the role of young males in teenage pregnancy outcome and prevention should be studied in the geographical regions of Uganda, since teenage pregnancy is often blamed on girls alone^18^,^19^. Globally, reduction of teenage pregnancy is a reproductive health target for sustainable development goal. Uganda is known to have policies that cater for better sexual reproductive health of teenagers. The presence of elevated levels of teenage pregnancy in some parts of the country should be investigated. An in-depth qualitative study is being conducted to investigate other determinants of the regional variation of teenage pregnancy in Uganda.
